# Detecting exacerbations using the Clinical COPD Questionnaire

**DOI:** 10.1186/1477-7525-8-102

**Published:** 2010-09-16

**Authors:** Jaap CA Trappenburg, Irene Touwen, Gerdien H de Weert-van Oene, Jean Bourbeau, Evelyn M Monninkhof, Theo JM Verheij, Jan-Willem J Lammers, Augustinus JP Schrijvers

**Affiliations:** 1 Julius Center for Health Sciences and Primary Care, University Medical Center Utrecht, Heidelberglaan 100, 3584 CX Utrecht, The Netherlands; 2 Respiratory Epidemiology and Clinical Research Unit, Montreal Chest Institute, McGill University Health Center, McGill University, 3650 St. Urbain Street, Montreal, Canada; 3 Department of Respiratory Medicine, University Medical Center Utrecht, Heidelberglaan 100, 3584 CX Utrecht, The Netherlands

## Abstract

**Background:**

Early treatment of COPD exacerbations has shown to be important. Despite a non-negligible negative impact on health related quality of life, a large proportion of these episodes is not reported (no change in treatment). Little is known whether (low burden) strategies are able to capture these unreported exacerbations.

**Methods:**

The Clinical COPD Questionnaire (CCQ) is a short questionnaire with great evaluative properties in measuring health status. The current explorative study evaluates the discriminative properties of weekly CCQ assessment in detecting exacerbations.

**Results:**

In a multicentre prospective cohort study, 121 patients, age 67.4 ± 10.5 years, FEV_1 _47.7 ± 18.5% pred were followed for 6 weeks by daily diary card recording and weekly CCQ assessment. Weeks were retrospectively labeled as stable or exacerbation (onset) weeks using the Anthonisen symptom diary-card algorithm. Change in CCQ total scores are significantly higher in exacerbation-onset weeks, 0.35 ± 0.69 compared to -0.04 ± 0.37 in stable weeks (p < 0.001). Performance of the Δ CCQ total score discriminating between stable and exacerbation onset weeks was sufficient (area under the ROC curve 0.75). At a cut off point of 0.2, sensitivity was 62.5 (50.3-73.4), specificity 82.0 (79.3-84.4), and a positive and negative predictive value of 43.5 (35.0-51.0) and 90.8 (87.8-93.5), respectively. Using this cut off point, 22 (out of 38) unreported exacerbations were detected while 39 stable patients would have been false positively 'contacted'.

**Conclusions:**

Weekly CCQ assessment is a promising, low burden method to detect unreported exacerbations. Further research is needed to validate discriminative performance and practical implications of the CCQ in detecting exacerbations in daily care.

## Background

Chronic Obstructive Pulmonary Disease (COPD) is a progressive chronic disease, characterized by an irreversible decline in lung function, exercise capacity and health status. The natural history of COPD is interrupted by exacerbations: episodes of worsening symptoms and signs, accelerating lung function decline [1,2] and responsible for decreased health related quality of life (HRQoL)[3,4], increased mortality [5,6] and health-care costs[7,8].

Irrespective of the definition of exacerbation used, the clinical diagnosis points to an acute clinical worsening that may necessitate a change in regular treatment[9]. Early identification and prompt treatment of exacerbations has shown to reduce exacerbation recovery time, while improving health related quality of life and reducing the risk of hospital admission[10]. Despite the importance of early treatment, several cohort studies have shown that a majority (East London 49%-54%[1,10,11], Canada 68%[12]) of exacerbations are not reported and most likely not treated. Although unreported exacerbations tend to be milder, these unreported exacerbations still have clinically relevant impact on HRQoL[12,13]. Therefore, event-based (based on use of healthcare services or treatment) definitions of exacerbations fail to capture all clinically important exacerbations. Symptom-based definitions, based on the increase of at least one key symptom for two consecutive days (dyspnea, sputum color, sputum volume[14]), are more likely to catch all exacerbations. However, this does not imply that these exacerbations will all be automatically reported in daily clinical routine. Symptom-based exacerbation definitions have shown to be a valid method of retrospective exacerbation identification in several research cohorts. In the meantime, there is insufficient evidence whether prospective daily symptom registration based on symptom-based exacerbations algorithms is effective in decreasing the amount of unreported exacerbations. In addition, it is uncertain whether patients would be compliant to long-term daily symptom registration and whether reporting would still depend on patients' responsibility to seek treatment and health care. Early studies on action plans aiming at early identification of exacerbations by patients and early treatment failed to show clinically relevant effects on healthcare utilization as well as on patient-reported outcomes, although these studies were all suffering from methodological limitations and were underpowered [15]. More recent studies have shown that the implementation of an action plan with self-administered prescription of antibiotic and prednisone had the potential to reduce physician visits [16,17] and hospital admissions[17].

Given the importance of early treatment, there is a need for new low-burden strategies to capture symptom based exacerbations. The Clinical COPD Questionnaire (CCQ) has shown to be a brief and useful tool to evaluate disease severity and response to treatment [18,19]. The CCQ was developed and validated in 2003 in order to measure health status in daily clinical practice and showed to have strong discriminative properties and responsiveness[19]. In this study, our aim was to determine the diagnostic value of weekly CCQ assessment to detect exacerbations. More specifically, the objective of the study was to assess the performance of weekly CCQ change in discriminating between stable and symptom-based exacerbation onset weeks, with respect to exacerbation severity. Accuracy in detecting exacerbations for weekly CCQ change was assessed using different cut off scores.

## Methods

### Design

Data were obtained from a pilot-study to develop an Action Plan for COPD patients which subsequently is used in a randomized clinical trial[19,20]. Between January and March 2008, COPD patients were recruited from inpatient (post-discharge) and outpatient clinics from the University Medical Hospital in Utrecht, six peripheral hospitals and five general practices. Patients were followed up for a period of six weeks (42 days).

### Study patients

Inclusion criteria were age over 40 years, dyspnea and/or chronic cough with or without sputum production, history of smoking (> 20 years of smoking or > 15 packyears), a post-bronchodilator FEV_1_/FVC of ≤ 0,7 according to the Global Initiative for Chronic Obstructive Lung Disease (GOLD) standards[21]. Patients with a primary diagnosis of asthma, cardiac disease or other major functionally limiting disease were excluded. Ethical approval was obtained from the Medical-Ethical Review Committee, and all patients gave their written informed consent prior to inclusion.

### Outcome measures

Patients were asked to record major and minor symptom deteriorations, according to Anthonisen, on a daily basis on a diary card [14]. Patients were instructed to fill in this diary at a fixed moment of the day; after their evening meal. The diary card consisted of major symptoms (dyspnea, sputum volume and sputum color), and minor symptoms (sore throat, fever, cough, common cold, wheezing). In addition, patients were instructed to note whether they contacted a physician or increased their inhalation medication, started corticosteroids or antibiotics. These contacts were subsequently labeled as respiratory and non-respiratory contacts. Major symptoms were scored when an increase was perceived. Minor symptoms were scored when they were present that day and not part of their normal symptom status. Patients were contacted by telephone by the investigators after the first 7 days to review their compliance and understanding of the daily assessments.

In addition, the Clinical COPD Questionnaire (CCQ)[19] was integrated in the diary card and assessed weekly. The CCQ is a self-administered questionnaire containing ten questions, divided into three domains: symptoms, mental and functional state. The questions are based on a 7-point scale where patients score a '0' when they are asymptomatic or have no limitation and a '6' when they are extremely symptomatic or completely limited. The final score is the mean of all ten items, and scores for the three domains can be calculated separately if required. High scores reflect poor health status. A week version and a 24 hour version exist of this questionnaire. The week version was used for the purpose of this study. Patients were asked to record their experiences during the last seven days. The CCQ was completed on day 'zero' and subsequently once every 7 days.

### Exacerbations

**Figure 1:**
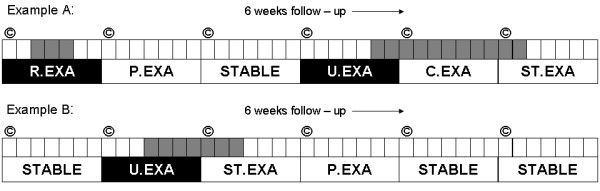
**Schematic example of two patients with a follow-up of 6 weeks with exacerbation days highlighted in grey**. Weeks are labelled based on the occurrence of an exacerbation. ^© ^= CCQ assessment. R.EXA = Recovered Exacerbation onset week, U.EXA = Unrecovered Exacerbation onset week; P.EXA = Post-Exacerbation week, STABLE = Stable week, C.EXA = Continued Exacerbation week, ST.EXA = Exacerbation Stop week.

A symptom-based definition was used to capture exacerbations. An exacerbation was defined as an increase in any two major symptoms, or increase in one major and the presence of at least one minor symptom for at least two consecutive days, according to Anthonisen et al [14]. Type 1 exacerbations were defined by an increase of dyspnea, sputum purulence and sputum volume; Type 2 when two of these symptoms increased; Type 3 when 1 symptom increased in addition to the presence of a minor symptom. Sputum purulence was scored as increased when either the sputum colour changed or sputum thickness increased that day. When patients enrolled in the study with an exacerbation, they were excluded from further analyses, because of the unknown time of onset. An event was considered reported if a patient reported respiratory symptom increase to a healthcare provider in an unscheduled telephone contact, physician or emergency room visit or was admitted to a hospital for an exacerbation. Rules were formulated to determine exacerbation onset. Since symptoms often fluctuate during and after exacerbations (10), it was stated that a new exacerbation could only take place after at least one stable week (or post-exacerbation week). Every week was coded as: a 'stable week' when no exacerbation took place in that week and the previous week (STABLE), a 'recovered exacerbation onset week' with recovery within that week (R.EXA), an 'unrecovered exacerbation onset week' without recovery within that week (U.EXA) a 'continuous exacerbation week' when an exacerbation continued after the onset week during this week (C.EXA), a 'stop week' week in which the exacerbation stopped (ST.EXA); and a 'post-exacerbation week' which is a stable week following an exacerbation (P.EXA) (figure 1). After a post-exacerbation week, it was possible to have a stable week or to have a new exacerbation week. An example is shown in figure 1.

### Statistical analysis

Data are presented as mean ± SD unless stated otherwise. All analyses were performed with SPSS software version 15.0. P < 0.05 was considered statistically significant in the analyses. Student's t-test and Mann Whitney test were used to compare baseline characteristics. For each week-interval the CCQ change (Δ-CCQ-score) is calculated by subtracting the CCQ score of the 'present' week from the CCQ score of the previous week. Differences in CCQ change for the different types of weeks was evaluated using the unpaired Student t-test. Discriminative performance of weekly CCQ change is analysed for three groups of exacerbations (recovered, unrecovered and all exacerbations) using receiver operating characteristic (ROC) curves to determine area under the curve (AUC) and 95% confidence interval. Contingency tables were made to calculate the following performance parameters for optimal cut-off points: sensitivity, specificity, positive predictive value (PPV) and negative predictive value (NPV) and overall accuracy. Spearman's rank-order correlation coefficient was used to determine association of change (Δ) in CCQ scores and the number of days between exacerbation onset and the post-week CCQ assessment.

## Results

### Patients

**Figure 2:**
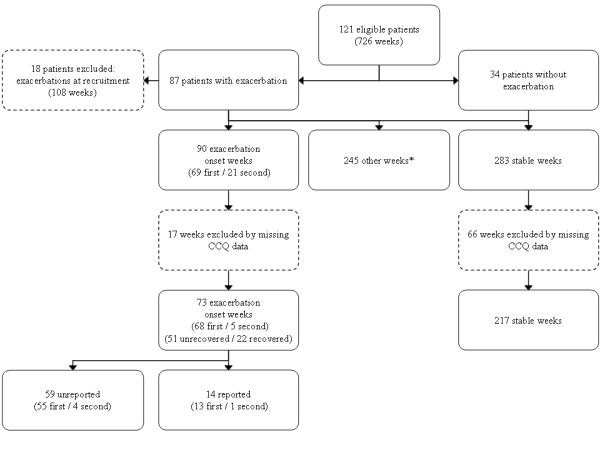
**Flow diagram showing number of patients, exacerbations and exacerbation weeks**. *: Exacerbation stop weeks, continued exacerbation weeks and post-exacerbation weeks.

One-hundred ninety-two (192) patients were recruited during the study period, of which 121 agreed to participate in the study (figure 2). Eighteen (18) patients were excluded because of exacerbation at enrolment. This resulted in a total of 103 patients eligible for analysis. Sixty-nine (69) patients (67%) experienced at least one exacerbation and 21 patients (20%) experienced two exacerbations, resulting in a total of 90 exacerbations. Delta-CCQ data were missing in 17 patients, therefore, data on 73 exacerbations could be analysed. Thirty-four (34) patients had no exacerbation. Baseline characteristics of patients with and without an exacerbation are shown in table 1. No statistically significant differences were seen, although patients with an exacerbation tended to be older, had more severe COPD and were more frequently included during hospital admission.

**Table 1:** Baseline characteristics

Baseline characteristics	Patients with an exacerbation	Patients without an exacerbation
No. of patients	69	34
Gender		
male n (%)	42 (60)	20 (58.8)
Age, yr	67 ± 9.8	65 ± 11.1
Current smoker n (%)	19 (27)	7 (21)
Smoking pack years (IQR)	37 (15-50)	37.5 (1-43.3)
FEV_1 _(% of predicted)	46.0 ± 18.4	51.1 ± 21.0^§^
FEV_1_/FVC	0.44 ± 0.13	0.47 ± 0.12
GOLD Classification of COPD n (%)		
stage I: mild	4 (5.8)	4 (11.8)
stage II: moderate	24 (34.8)	10 (29,4)
stage III: severe	27(39.1)	16 (47,1)
stage IV: very severe	14 (20.3)	4 (11.8)
MRC dyspnea	3 (2-4)	3 (2-4)
Contact reason at inclusion n (%)		
regular check-up	36 (51.4)	13 (38.2)
emergency department	2 (2.9)	0 (0)
during hospitalisation	14 (20)	5 (14.7)
check-up after hospitalisation	1 (1.4)	1 (2.9)
during pulmonary rehabilitation	16 (22.9)	15 (44.1)

FEV_1_: Forced Expiratory Volume in 1 second; GOLD: Global inititiative for Chronic Obstructive Lung Disease; MRC: Medical research counsel. Data are presented as mean ± SD or median (interquartile range) or number with percentage in parenthesis. ^§ ^p < 0.05

### CCQ discriminative performance

**Figure 3:**
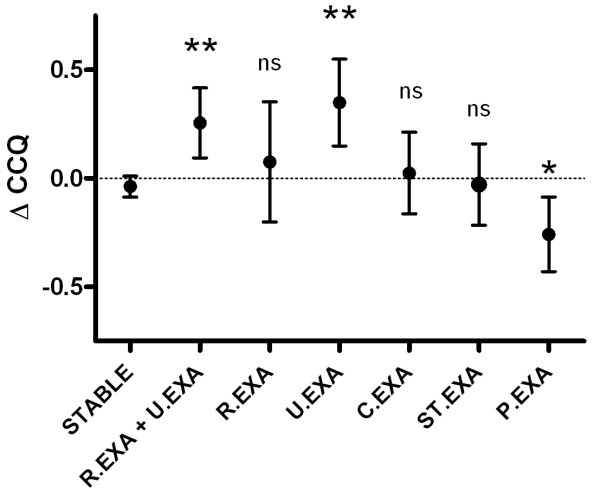
**Change in CCQ-total score for different exacerbation related week types compared to stable weeks**. R.EXA = Recovered Exacerbation week, P.EXA = Post-Exacerbation week, STABLE = Stable week, U.EXA = Unrecovered Exacerbation week, C.EXA = Continued Exacerbation week, ST.EXA = Exacerbation Stop week, Unpaired t-test; *: p < 0.01, **: p < 0.001.

Figure 3 shows change in CCQ-total scores for different types of weeks, irrespective of exacerbation type (Anthonisen I -, II - and III). Compared to stable weeks (Δ-CCQ-score: -0.04, 95%CI: -0.09 to 0.01), mean change in CCQ scores were significantly higher in all (R.EXA + U.EXA) exacerbation onset weeks (Δ-CCQ-score: 0.26, 95%CI: 0.09 to 0.42 p < 0.001), unrecovered (U.EXA) exacerbation onset weeks (ΔCCQ-score: 0.35, 95% CI: 0.15 to 0.55, p < 0.001), but not in the recovered (R.EXA) exacerbation onset weeks (Δ-CCQ-score: 0.08, 95%CI: -0.20 to 0.35, p = 0.19). In post-exacerbation weeks, the mean change in CCQ-total score decreased significantly (Δ-CCQ-score: -0.26, 95% CI: -0.43 to -0.09, p = 0.001).

Results show that CCQ change is associated with the type of exacerbations, i.e., types 1 and 2 but not type 3, as reflected in all CCQ domains (table 2). Since the CCQ is taken weekly, the median detection delay (time between post CCQ assessment and exacerbation onset) is 5 days (IQR 3-6). No association was found between detection delay and weekly CCQ change scores (spearman's rho = 0.12, p = 0.29).

**Table 2:** Changes in weekly CCQ scores for unrecovered exacerbations according to exacerbation severity types [14]

	Δ-CCQ total score	Δ-CCQ symptoms	Δ-CCQ functional state	Δ-CCQ mental state
Type 1: 3 symptoms (n = 20)	0.56 (0.20 - 0.92)	0.50 (0.46 - 0.95)	0.39 (0.03 - 0.74)	0.52 (0.18 - 1.03)
Type 2: 2 symptoms (n = 24)	0.29 (0.03 - 0.56)	0.14 (-0.15 - 0.43)	0.25 (-0.18 - 0.68)	0.18 (-0.09 - 0.46)
Type 3: 1 symptom (n = 29)	0.01 (-0.22 - 0.25)	0.07 (-0.18 - 0.32)	-0.05 (-0.35 - 0.26)	0.16 (-0.13 - 0.45)
All exacerbations (n = 73)	0.26 (0.09 - 0.42)	0.21 0.03 - 0.39)	0.18 (0.03 - 0.38)	0.27 (0.07 - 0.47)

Data are presented as mean (95% confidence interval).

**Figure 4:**
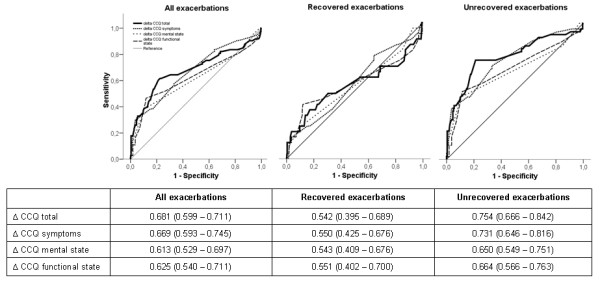
**Receiver operating curves for all exacerbations, recovered exacerbations and unrecovered exacerbations**. Table: Area under the ROC curve for the different CCQ measures. Data are presented as mean (95% confidence interval).

Figure 4 presents the ROC curves assessing accuracy of the weekly Δ-CCQ score in discriminating between stable and exacerbation weeks. Highest area under the ROC curve is observed in unrecovered exacerbation types. Within this group, AUC was 0.75 (95% CI 0.67 to 0.84).

### CCQ accuracy for different cut off scores

Estimates of discriminative performance of the CCQ total score are calculated for four cut off points on the ROC curve; 0.1, 0.2, 0.3 and 0.4 points (Table 3). Lower cut-off points were associated with more false positives and less false negatives. For the detection of unrecovered exacerbations, the probability of correctly detecting patients with an exacerbation onset is moderate for all cut off scores, with an optimal sensitivity of 72.9% at the lowest cut off of 0.1 points. Highest probability of correctly excluding patients without the condition is seen at a cut off of 0.4 points with an optimal specificity of 93.1% but resulting in a poor sensitivity (39.6%). Overall correct classification rate (accuracy) was higher when Δ CCQ cut off score increased. Within all cut off points, the proportions of captured exacerbations (true-positives) not reported to a healthcare provider were similar, ranging from 68 to 74%, with a comparable distribution in exacerbation severity.

**Table 3:** Discriminative properties of four ΔCCQ total score cut off points for unrecovered exacerbations

	Δ CCQ total score in unrecovered exacerbations			
	**Cut off** **0.1 points**	**Cut off** **0.2 points**	**Cut off** **0.3 points**	**Cut off** **0.4 points**

Sensitivity, %	72.9 (60.5 - 82.8)	62.5 (50.3 - 73.4)	47.9 (36.4 - 58.9)	39.6 (29.2 - 49.1)
Specificity, %	69.1 (66.1 - 71.3)	82.0 (79.3 - 84.4)	87.6 (85.0 - 90.0)	93.1 (90.8 - 95.2)
PPV, %	34.3 (28.5 - 39.0)	43.5 (35.0 - 51.0)	46.0 (35.0 - 56.6)	55.9 (41.3 - 69.3)
NPV, %	92.0 (88.4 - 94.9)	90.8 (87.8 - 93.5)	88.4 (85.8 - 90.8)	87.4 (85.3 - 89.4)
Overall accuracy, %	69.8 (65.3 - 73.4)	78.5 (74.1 - 82.4)	80.4 (76.2 - 84.4)	83.4 (79.6 - 86.8)
True positive, n	35	30	23	19
False positive, n	67	39	27	15
Unreported, n (% of TP)	26 (74)	22 (73)	16 (70)	13 (68)
Exacerbation type I, n (% unreported)	8 (31)	5 (23)	4 (25)	4 (31)
Exacerbation type 2, n (% unreported)	11 (42)	10 (45)	6 (37.5)	5 (38)
Exacerbation type 3, n (% unreported)	7 (27)	7 (32)	6 (37.5)	4 (31)

Overall accuracy = (number of true-positives+ true negatives)/(number of true positives + true negatives + false positives + false negatives). PPV: Positive Predicted Value. NPV: Negative Predicted Value. TP: True Positive. Data are presented as value or ratio (95% confidence interval).

## Discussion

This explorative study shows that in exacerbation onset weeks, CCQ scores were significantly increased compared to stable weeks, especially for unrecovered exacerbations. This is in line with a Canadian cohort which also indicated immediate CCQ deteriorations following identification of an exacerbation [22]. In our study, the performance of CCQ change to discriminate between stable weeks and exacerbation onset weeks was acceptable. The highest discriminative power (AUC 0.75) was seen for the CCQ total score in the unrecovered exacerbation group. These results indicate that weekly CCQ assessment is able to detect formerly unidentified but important exacerbations. However, also a substantial number of type I (false positives) and type II error (false negatives) were present. Exploration of this ROC curve showed that overall sensitivity and PPV are relatively disappointing and substantially vary between different cut offs. Highest cut off scores result in higher overall classification rates (accuracy). Lowering the cut off score (0.1 points) is associated with the highest but still moderate sensitivity and NPV of 72.9% and 92% respectively. However, there is a marked increase in false positives. In contrast, the highest cut off of 0.4 points resulted in decreasing the false positive rate (specificity 93.1%), but is less accurate in correctly detecting exacerbation onset (sensitivity 39.6%) Interpretation and extrapolation of the results in answering whether weekly CCQ assessment is or is not a useful screening tool and identifying the most optimal trade-off point between sensitivity and specificity for detecting unreported exacerbations need to be done with caution.

The present study has several limitations. First, the examination of a relatively small prospective group of 103 consecutive patients followed up for only 6 weeks resulted in 90 exacerbations which is equal to an annual rate of 7.5 per patient-year. This is a relatively high rate and could have affected generalizability of our results since PPV and NPV are strongly related to prevalence[23]. The high event-rate can be explained because all patients were simultaneously followed-up in the same 6-week winter period in which exacerbations have shown to be ~ 50% more likely than in other seasons[4,24]. Also the relative high proportion of patients included immediately after hospitalisation might have contributed to a higher exacerbation rate. Another consequence of the very short study follow-up of 6 weeks without a run-in period (allowing to include only stable patients), 17 exacerbation onset weeks (19%) and 66 (23%) stable weeks were excluded because it was not possible to assess CCQ change (exacerbation onset before inclusion, or onset in the sixth week). Therefore, diagnostic accuracy estimates showed considerable statistical uncertainty (wide 95% confidence intervals). Results of this explorative study need to be validated in larger studies with a preferable follow-up of at least one year. A run-in period including giving adequate feedback could potentially enhance patient compliance of completing questionnaires, subsequently decreasing the amount of (partly) missing or invalid data. Furthermore, we observed that reinforcement by telephone (at 7 days) has the potential to increase understanding and compliance. Future studies with daily symptom registrations should preferably incorporate these calls more frequently, especially in the early stage of follow-up (i.e. at 1 and 2 months).

Secondly, operative characteristics of this test rely heavily on the reference standard used. Despite inconsistency in the literature regarding methods to define exacerbation[25], we decided to use the symptom-based exacerbation algorithm of Anthonisen[14]. This algorithm has been widely used to assess symptom-based exacerbations and has shown to capture more exacerbations than strictly depending on event-based definitions[11,12]. Nevertheless, diagnostic accuracy for both exacerbation presence as well as severity categories has never been properly validated. Anthonisen's classification never intended to establish the severity of exacerbation using type of exacerbations (type 1, 2 and 3) but rather which patient could benefit from being treated with antibiotics.

Thirdly, discriminative performance was assessed comparing weekly CCQ change between exacerbation onset weeks and all stable study weeks. This means that stable weeks' assessment included both outcomes of patients with and without an exacerbation in the study period. It would have been favourable to perform paired analysis by comparing CCQ change in exacerbation onset weeks with stable periods within the same patient. This method could also determine individual bandwidths of non-exacerbation related CCQ variations in stable periods. Identification of CCQ change outcome beyond normal week to week variations might increase the likelihood of correct exacerbation detection. This obviously needs longer follow-up including an adequate run-in period. In addition, it can be expected that not all patients will fully recover from an exacerbation in terms of CCQ scores[22]. Longer follow-up enables evaluation of the CCQ across several (recurrent) exacerbations including the possibility to account for non-recovery and individually adjusted baseline levels. It needs to be emphasized that including multiple exacerbations in the analysis demands accounting for dependency on the patient level and the inherent correlation of paired data. In our analysis the impact of multiple-events is marginal since only 5 of the 73 exacerbation weeks were recurrent. Future studies assessing binary classifications (ROC-curves) to detect exacerbations should preferably account for multiple events. ROC curves and according discriminative properties for (partially) paired data should be statistically combined and plotted resulting in a combined AUC with corresponding confidence interval (ref vergara 2008, Metz 1998).

Although this study has several limitations, it adds to current knowledge, being the first study exploring the diagnostic value and practical implications of a low-burden (weekly) and easy-to-complete screening instrument for capturing exacerbations, both reported and unreported. To draw conclusions on the usefulness of weekly CCQ assessments and to define a cut off point as the most optimal trade-off, it is essential to reconsider what are the most important discriminative properties, necessary for this screening tool's optimal clinical utility. For example, the implications of weekly CCQ assessments with a cut off of 0.2 points as a screening tool in the current population in a winter period were as follows: 30 exacerbations would have been detected while only 8 exacerbations were reported (and presumably treated) without weekly CCQ monitoring. The additional 22 correctly detected (but unreported) exacerbations (of which 5 were severe) can be considered as a clinical benefit of the CCQ assessment. However, to be able to detect 22 additional exacerbations, 39 exacerbations would have been incorrectly (false positive) identified and possibly contacted. Still, cost-benefit ratios of verification of (true) exacerbations by contacting patients strongly depend on the technical implementation of screening and methods of contacting patients. If these costs are acceptable, it may be worth contacting 39 patients to be able to capture 22 unreported exacerbations. Detecting exacerbations using weekly assessment results in delayed detection varying between 1-6 days. Although little is known on how early early detection should be, we believe that for obvious reasons it remains preferable to detect (some) exacerbations with a 6-day delay instead of not detecting/treating.

Weekly monitoring of the CCQ appears to be a strictly passive method to enhance detection of exacerbations. Nevertheless, this does not necessarily compete with other methods aiming at early detection and treatment of exacerbations by enhancing self-management, for example by using action plans[15,26]. If weekly CCQ monitoring (regardless of the practical procedure) was implemented, this could actually also reinforce self-management behavior. Confronting patients with monitoring outcome and linking this with positive and negative self-management experiences and decisions could enhance self efficacy which is an important predictor of behaviour change [27,28]. In the current study, both symptom-based exacerbation identification as well as the CCQ were assessed using a daily paper diary. With regard to the relatively high amount of missing CCQ data in our study, future attempts should also incorporate methods to enhance compliance, such as reminders and possibilities to provide feedback or support. New developments in the field of telemonitoring and e-health have created opportunities to monitor COPD patients on a daily basis. Although this creates a fundamental technical starting point, also for low burden weekly CCQ monitoring, evidence on effects of telemonitoring is still relatively scarce [29].

## Conclusions

CCQ monitoring is a promising low burden method to detect a substantial proportion of unreported exacerbations. Despite the relatively high number of false positives, we believe this exploration is important and promising in view of the disturbingly high incidence of unreported and subsequently untreated exacerbations. Therefore, to confirm the performance of CCQ monitoring in discriminating between normal day to day variations and exacerbations, large diagnostic studies are needed with follow-up of at least one year covering both the seasonality and clustered aspect of exacerbations. This would enable intra-individual identification of periods in which CCQ changes are beyond the bandwidth of non-exacerbation related CCQ variations in stable periods. Furthermore different screening technologies should be evaluated for their daily utility and efficiency for different CCQ cut off scenarios. Finally, if both the test and the technology are well established, follow-up studies to quantify the effect of routine monitoring on patient outcome should be evaluated using randomized trials.

## Competing interests

The authors declare that they have no competing interests.

## Authors' contributions

All co-authors have read and approved the final manuscript. JCAT - leading of development of the study conceptualisation, design, refining of protocol and write up for publication. IT - major contribution to refining and substantial contribution to writing up of the protocol for publication. GHWO - major input into study conceptualisation, design and protocol publication. EMM - contribution to development of study design, statistical issues, contribution to protocol publication. JB - expert respiratory input contribution to study conceptualisation and refining on outcome measures, statistical issues and substantial contribution to writing up of the protocol for publication. TV/JWJL/AJP - contribution to study and intervention development and contribution to protocol publication.
